# Determinants of early initiation of breastfeeding in Amibara district, Northeastern Ethiopia: a community based cross-sectional study

**DOI:** 10.1186/s13006-016-0067-8

**Published:** 2016-04-08

**Authors:** Misgan Legesse Liben, Ebrahim Mohammed Yesuf

**Affiliations:** Department of Public Health, College of Medical and Health Sciences, Samara University, Samara, Afar Ethiopia

**Keywords:** Early breastfeeding initiation, Agro-pastoral, Amibara, Afar, Ethiopia

## Abstract

**Background:**

Early initiation of breastfeeding has lifetime benefits for the mother and the child. It has a positive impact on the duration of exclusive breastfeeding. Hence, the initiation of breastfeeding within the first hour of life lays the foundation for optimal breastfeeding. This study aimed to assess early and timely initiation of breastfeeding practices and associated factors among mothers of children aged less than 24 months in Amibara district of Northeast Ethiopia during April 2015.

**Methods:**

A quantitative community based cross-sectional study was employed on 407 mothers of children aged less than 24 months in Amibara district. Descriptive statistics, binary and multivariable logistic regression analysis were employed to identify the factors associated with early initiation of breastfeeding. The strength of the association was measured by odds ratio, and *p*-value < 0.05 was considered statistically significant.

**Results:**

Three hundred eighty one (94.5 %) of the respondents had ever breastfed their index child. Of those who had ever breastfed, 151, 39.6 % (95 % Confidence Interval [CI] 35.0 %, 45.0 %) of mothers initiated breastfeeding within 1 h after birth. In multivariable logistic regression analysis mothers living in urban areas (Adjusted Odds Ratio [AOR] 3.8; 95 % CI 2.32, 6.06) and who attended formal education (AOR 2.0; 95 % CI 1.21, 3.46) were associated with increased odds of early initiation of breastfeeding. The factors associated with decreased odds of timely initiation of breastfeeding were caesarean section delivery (AOR 0.46; 95 % CI 0.22, 0.97) and mothers with two or three children (AOR 0.59; 95 % CI 0.35, 0.99).

**Conclusion:**

This study showed that four in ten infants were breastfed within the first hour after birth. Therefore, providing proper support and guidance of health professionals during cesarean section delivery and breastfeeding education programs at the village level for girls and young women without formal education are important interventions to promote early initiation of breastfeeding in the study area.

## Background

The World Health Organization (WHO) and United Nations Children’s Fund (UNICEF) recommend exclusive breastfeeding for children up to 6 months of age, and to nourish them with appropriate complementary foods and continued breastfeeding from 6 months until 24 months or beyond. Exclusive breastfeeding is described as, an infant’s breast milk consumption without supplementation of any type of foods or drinks, except for vitamins, minerals and necessary medications for the first 6 months [1]. It is associated with reduced child morbidity [2]. As a result nursing mothers are encouraged to initiate breastfeeding early, and to feed their infants only with breast milk in the first 6 months.

Early initiation of breastfeeding is the initiation of breastfeeding within 1 h after birth [3]. It has lifetime benefits for the mother and the child. Mothers benefit from early suckling because it stimulates breast milk production and facilitates the release of oxytocin, which helps the contraction of the uterus and reduces postpartum hemorrhage. Moreover, early initiation of breastfeeding helps infants to get the first breast milk, known as colostrum. Colostrum is highly nutritious and has antibodies that protect the newborn from disease [4].

Early initiation of breastfeeding facilitates emotional bonding of the mother and the newborn, and has a positive impact on the duration of exclusive breastfeeding. Prelacteal feeding has a negative impact on breastfeeding [5] and initiation of breastfeeding in the first hour of life lays the foundation for optimal breastfeeding [6]. Optimal breastfeeding up to 2 years of age has the potential to prevent 800,000 deaths of under-five age children annually [7]. Of these 22.3 % of neonatal deaths could be prevented if all children had been breastfed in the first hour of life [8]. Therefore, interventions that will promote early initiation of breastfeeding can result in significant reduction in neonatal mortality.

In order to reduce barriers and practices that prevent appropriate and early exclusive breastfeeding, Ethiopia has developed the National Infant and Young Child Feeding (IYCF) Guideline. The guideline aims to achieve optimal breastfeeding practices through promotion of early initiation of breastfeeding. However, a wide range of infant feeding malpractices are documented even after implementation of the Ethiopian National IYCF Guideline in 2004 [6]. Therefore, this study was conducted in Amibara district to assess early initiation of breastfeeding practice and associated factors among mothers of children aged less than 24 months. The findings of this study will be vital for health service providers, policy makers and program managers to design intervention strategies that can promote early initiation of breastfeeding in the district.

## Methods

### Study design and participants

A community based cross sectional study was conducted in Amibara district during April, 2015. Amibara district is one of the six districts of Zone Three, which is located 250 kms northeast of Addis Ababa. Amibara district is the main livestock productive area, which is bordered on the south by Awash Fentale, on the west by the Awash River, on the northwest by the Administrative Zone Five, on the north by Gewane, on the east by the Somali Region and on the southeast by Oromia region. It is found in Awsa and Gewane agro-pastoral zone. This agro-pastoral zone is characterized by irrigated and rain-fed crop production combined with livestock rearing. Cotton and irrigation based crop production are mainly practiced in this district.

There are eighteen kebeles (the smallest administrative units next to district in Ethiopia) under Amibara district; three urban and fifteen rural kebeles. Based on the 2007 Census population projection, the district has a total population of 76, 649. Of these 37.7 % are urban inhabitants, 51.96 % agro-pastoralists and 10.34 % pastoralists. Children under 2 years of age are estimated to be 2.5 % of the total population and average family size of the households to be 4.5 people. There are three health centers and eighteen health posts in the district [9].

A total of 407 mother-child pairs were calculated using a single population proportion formula;

$$ \mathrm{n}=\frac{{\left(\mathrm{z}\frac{\upalpha}{2}\right)}^2\mathrm{p}\left(1-\mathrm{p}\right)}{{\mathrm{d}}^2} $$

Where:

n = required sample size

$z\frac{\alpha }{2}$ = critical value for normal distribution at 95 % confidence level which equals to 1.96 (z value at alpha 0.05)

p = prevalence of early initiation of breastfeeding

d = an absolute precision (d = 0.05)

Assumptions:

5 % margin of error, 95 % confidence level, 59.6 % prevalence of early initiation of breastfeeding in Afar region of Ethiopia [10], and 10 % for non-response.

### Sampling procedure and technique

The sampling procedure was started from the stratification of the fifteen kebeles as rural and the three kebeles as urban. Out of the eighteen kebeles, two urban and three rural kebeles were randomly selected using lottery method. About 38 % (37.7 %) of mothers of children aged less than 24 months live in urban areas and the remaining 62.3 % live in rural areas. Therefore, based on population proportion, 254 were taken from rural areas and 153 from urban areas.

Then the sample size was proportionally allocated to each selected kebeles. At the time of survey, from each household unit one eligible mother who had a biological child aged less than 24 months was selected. If there were more than one mother with children aged less than 24 months in one household unit, one mother with the youngest child was selected. From mothers who had two children aged less than 24 months, the youngest child was selected as the reference. If mothers had twin children aged less than 24 months, one child was selected by lottery.

### Data collection process and instrument

Data were collected using a pre-tested, structured and interviewer administered questionnaire adapted from the Ethiopian Demographic and Health Survey [10] and the National Nutrition Survey Questionnaire [11]. The adapted questionnaire was modified and contextualized to fit the local situation and the research objective. The questionnaire was prepared first in English and translated in to Qafar’af, then back to English to check for consistency. The Qafar’af version of the questionnaire was used to collect the data. The data was collected by five preparatory school graduates. The data collectors and the supervisors (two BSc nurses) were trained for 2 days by the principal investigator on the study instrument, consent form, how to interview and data collection procedure.

### Study variables

In this study the dependent variable was ‘early initiation of breastfeeding practice’ among mothers of children aged less than 24 months. Early initiation of breastfeeding is the initiation of breastfeeding within 1 h of birth [3]. Initiating breastfeeding within 1 h of birth was coded as ‘1’, while initiating breastfeeding after 1 h of birth was coded as ‘0’ for regression analysis. The independent variables were maternal characteristics (age, religion, ethnicity, educational status, marital status, parity), household characteristics (area of residence either rural or urban, household head, family size), paternal educational status, sex of the index child, birth order of the index child, antenatal care utilization, place of delivery, mode of delivery, postnatal care utilization, prelacteal feeding and colostrum avoidance practices.

### Data quality control

Preparatory school graduates who can speak the local language were recruited as data collectors. The questionnaire was pretested on 21 mothers (5 % of the sample size) having at least one child aged less than 24 months before the initiation of the study. These mothers were sampled from two kebeles which were not included in the research. The pretest was done to ensure clarity, wordings, logical sequence and skip patterns of the questions. Then the pretest amendments on the questionnaire were made accordingly. The supervisors checked the day to day activities of data collectors regarding the completion of questionnaires, clarity of responses and proper coding of the responses.

### Statistical analysis

The data were checked for completeness and consistencies. Data were also cleaned, coded and entered into EpiData version 3.02, then exported to SPSS version 20 statistical package for analysis.

The Crude Odds Ratios (COR) with 95 % confidence interval were estimated in the binary logistic regression analysis to assess the association between each independent variable and the outcome variable, and to select candidate variables for the multivariable logistic regression analysis. Variables with *p*-value < 0.3 in the binary logistic regression analysis were considered in the multivariable logistic analysis.

The Hosmer-Lemeshow goodness-of-fit with enter procedure was used to test for model fitness. Adjusted Odds Ratio with 95 % confidence interval was estimated to assess the strength of the association. In this study, multicollinearity among independent variables was detected using the standard errors for regression coefficients. Variables with *p*-value < 0.05 in the multivariable logistic regression analysis were considered as significant and independent predictors of early initiation of breastfeeding.

### Ethical considerations

The study was approved by the Ethical Review Committee (ERC) of Samara University. An official letter was written from Samara University to Amibara District Administration Office. Then permission and support letter was written to each selected kebeles. The participants enrolled in the study were informed about the study objectives, expected outcomes, benefits and the risks associated with it. A signed written consent was taken from the participants before the interview. Illiterate mothers were consented with their thumb print. Confidentiality of responses was maintained throughout the study.

## Results

### Socio-demographic characteristics of the participants

A total of 403 mother-child pairs were included in the study with a response rate of 99 %. About 75 % of the mothers were in the age group of 20–34 years. More than half of mothers (56.8 %) were Muslims, 72.5 % were currently married and 37 (9.2 %) of mothers never married (single). Only 22.8 % of mothers were household heads, and nearly 57 % had five and more family members (Table 1). Regarding maternal and child health (MCH) service utilization, the majority of mothers had attended antenatal care visits (75.9 %), 86.4 % of mothers had spontaneous vaginal delivery and 65 % had attended postnatal care visits (Table 2).

**Table 1:** Socio-demographic characteristics of mothers of children aged less than 24 months in Amibara district, North Eastern Ethiopia, April 2015

Variables	Frequency (*n*)	Percent (%)
Age of mother (in year) (*n* = 403)		
< 20	21	5.2
20–34	302	74.9
> 34	80	19.9
Mean (± SD) & median age of mother	28.94 (±6.29) & 29.00 years	
Ethnicity (*n* = 403)		
Afar	208	51.6
Amhara	106	26.3
Oromo	54	13.4
Tigray	35	8.7
Religion of mother (*n* = 403)		
Muslim	229	56.8
Orthodox	119	29.6
Protestant	55	13.6
Maternal education status (*n* = 403)		
No formal education	131	32.5
Formal education	272	67.5
Maternal marital status (*n* = 403)		
Currently married	292	72.5
Unmarried^a^	111	27.5
Paternal educational status (*n* = 366)		
No formal education	202	55.2
Formal education	164	44.8
Head of the household (*n* = 403)		
Mother of the index child	92	22.8
Fathers of the index child	311	77.2
Parity (*n* = 403)		
Primipara	103	25.6
Multipara	300	74.4
Sex of the index child (*n* = 403)		
Male	203	50.4
Female	200	49.6
Age of the child (in month) (*n* = 403)		
< 6	64	15.9
6–11	60	14.9
12–17	102	25.3
18–23	177	43.9
Mean (± SD) & median age of child	14.50 (± 7.36) & 15.00 months	
Family size (*n* = 403)		
2	30	7.5
3–4	142	35.2
≥ 5	231	57.3

^a^Single, divorced and widowed

**Table 2:** Distribution of mothers based on maternal and child health service utilization in Amibara district, North Eastern Ethiopia, April 2015

MCH service utilization	Frequency	Percent (%)
Antenatal care (ANC) visit (*n* = 403)^a^		
Yes	306	75.9
No	97	24.1
Number of ANC visit (*n* = 306)		
1	20	6.5
2–3	122	39.9
≥ 4	164	53.6
Counseling on breastfeeding at ANC visit (*n* = 306)		
Yes	248	81.0
No	58	19.0
Delivery place (*n* = 403)		
Home	159	39.5
Health institution	244	60.5
Delivery Attendant(*n* = 403)		
Health professional	252	62.5
Traditional birth attendant	139	34.5
Self-care/No one	12	3.0
Mode of delivery (*n* = 403)		
Vaginal delivery	348	86.4
Caesarean section	55	13.6
Postnatal care (PNC) visit (*n* = 403)^a^		
Yes	262	65.0
No	141	35.0
Counseling on breastfeeding at PNC visit (*n* = 262)		
Yes	218	83.2
No	44	16.8

^a^at least one visit

### Early initiation of breastfeeding practice and associated factors

Three hundred eighty one (94.5 %) of the respondents had ever breastfed their index child. Of those who had ever breastfed, 151, 39.6 % (95 % CI 35.0 %, 45.0 %) mothers initiated breastfeeding within 1 h after birth.

Binary logistic regression analysis showed that residence, household headship, maternal educational and marital status, parity, mode of delivery and birth order were statistically associated with early initiation of breastfeeding at *p*-value < 0.05 (Table 3). However, maternal age, ethnicity, religion, family size, prelacteal feeding and husband educational status were not significantly associated with early initiation of breastfeeding at *p*-value < 0.3 in the binary model. Thus, these variables were not included in multivariable regression model.

**Table 3:** Binary and multivariable logistic regression analysis showing factors associated with early initiation of breastfeeding among mothers of children aged less than 24 months in Amibara district, April 2015

Variables	Early initiation of breastfeeding *n* (%)	COR (95 % CI)	AOR (95 % CI)
Residence			
Urban	81 (55.9)	3.0 (1.95,4.62)^a^	3.8 (2.32,6.06)^a^
Rural	70 (29.7)	1	1
Antenatal care visit			
Yes	110 (37.8)	0.73 (0.45,1.17)	0.58 (0.32,1.05)
No	41 (45.6)	1	1
Maternal education			
No formal education	35 (28.7)	1	1
Formal education	116 (44.8)	2.0 (1.27,3.20)^a^	2.0 (1.21,3.46)^a^
Marital status of mothers			
Currently married	124 (44.0)	1	1
Unmarried	27 (27.3)	0.48 (0.29,0.79)^a^	0.76 (0.40,1.42)
Household head			
Mother of the index child	23 (28)	0.52 (0.31,0.89)^a^	0.69 (0.36,1.32)
Fathers of the index child	128 (42.8)	1	1
Place of delivery			
Home	66 (43.1)	1.28 (0.84,1.94)	0.65 (0.38,1.11)
Health institution	85 (37.3)	1	1
Parity			
Primipara	29 (30.9)	0.6 (0.37,0.99)^a^	1.57 (0.44,5.59)
Multipara	122 (42.5)	1	1
Sex of the index child			
Male	81 (42.4)	1.26 (0.84,1.91)	1.49 (0.94,2.30)
Female	70 (36.8)	1	1
Birth order of the index child			
1	28 (30.1)	0.44 (0.26,0.77)^a^	0.33 (0.08,1.28)
2–3	53 (36.3)	0.59 (0.37,0.94)^a^	0.59 (0.35,0.99)^a^
≥ 4	70 (49.3)	1	1
Colostrum avoidance			
No	68 (43.6)	1	1
Yes	83 (36.9)	0.76 (0.49,1.15)	0.66 (0.41,1.05)
Mode of delivery			
Cesarean section	12 (22.6)	0.40 (0.20,0.79)^a^	0.46 (0.22,0.97)^a^
Vaginal	139 (42.4)	1	1

*COR* crude odds ratio, *AOR* adjusted odds ratio, *CI* Confidence Interval. ^a^Statistically significant variables at *p* < 0.05. Hosmer-Lemeshow goodness-of-fit = 0.184

In multivariable logistic regression analysis living in urban places, maternal attendances of formal education, giving birth by caesarean section and birth order remained statistically significant factors associated with early initiation of breastfeeding (Table 3). Mothers living in urban areas were 3.8 times (AOR 3.8; 95 % CI 2.32, 6.06) more likely to initiate breastfeeding within 1 h of birth. Compared to mothers who had no formal education, mothers who attended formal education were two times (AOR 2.0; 95 % CI 1.21, 3.46) more likely to initiate breastfeeding within 1 h of birth. Mothers who had delivered their child by caesarean section (AOR 0.46; 95 % CI 0.22, 0.97) were less likely to initiate breastfeeding within 1 h of birth as compared to those who gave birth vaginally. Mothers with two or three children were less likely (AOR 0.59; 95 % CI 0.35, 0.99) to breastfeed their child within 1 h of birth compared to mothers with four or more children.

## Discussion

This study aimed to determine the prevalence of and the factors associated with early initiation of breastfeeding. The prevalence of early initiation of breastfeeding was 39.6 % (95 % CI 35.0 %, 45.0 %). In addition, multivariable logistic regression analysis showed that living in urban places and maternal attendances of formal education were associated with increased odds of early initiation of breastfeeding. In contrast caesarean section delivery and birth order were associated with decreased odds of early initiation of breastfeeding.

Although the WHO and Ethiopian IYCF guidelines recommend that all newborns should start breastfeeding immediately after birth, the present study showed that four children in every ten were benefited from early initiation of breastfeeding in Amibara district. This is the same as the prevalence of early initiation of breastfeeding in Axum town (41.6 %) [12]. However, it is lower than the prevalence of early initiation of breastfeeding in Afar Regional state (59.6 %) [10], Goba district (52.4 %) [13] and Raya Kobo district (71.7 %) [14]. This difference could be due to the different livelihood of the mothers, infant feeding styles and socio-cultural practices across the communities.

Mothers who live in urban areas were 3.8 times more likely to initiate breastfeeding within 1 h of birth compared to rural mothers. This could be explained because the mothers who live in urban places might have access to different information sources on breastfeeding including media. On the other hand rural mothers might not have access to such information sources. Similarly, in Goba district, urban mothers were three times more likely to initiate breastfeeding early as compared to their rural counterparts [13]. In contrast, rural mothers were more likely to initiate breastfeeding early as compared to urban mothers in Saudi Arabia [15].

Compared to mothers who had no formal education attendances, mothers who attended formal education were twice as likely to initiate breastfeeding within 1 h of birth. In Goba district, mothers who had formal education were also more likely to initiate breastfeeding with in the first hour after delivery as compared to those mothers who had no formal education [13]. This is similar to a report from India [16]. This might be because the mothers who had attended formal education might get essential information on proper breastfeeding practices in the school setup, and may also read breastfeeding promotion materials.

Mothers who had delivered their child by caesarean section were less likely to initiate breastfeeding within 1 h of birth as compared to those who had vaginal delivery. Similarly, cesarean section is a barrier to early breastfeeding initiation in Saudi Arabia [15], India [16] and Nepal [17]. This might be explained because mothers who deliver their child through cesarean section, take longer to recover from the effects of the anesthesia. This might cause a longer delay in making their first contact with their infant, and mothers might also find it difficult to achieve comfortable breastfeeding positions. In addition, infants born by cesarean section might have respiratory distress. Hence, they are more likely to be taken to a newborn intensive care unit and physically separated from their mother.

Birth order of the index child was significantly associated with early initiation of breastfeeding. In Amibara district, mothers were more likely to breastfed their children within 1 h after birth as the birth order increases. There was similar report from Nepal [18]. This might be due to the reason that women may update their beliefs about the benefits of breastfeeding with each birth. For example, a mother who found breastfeeding to be safer and cheaper for a first-born child may decide to breastfeed a subsequent child early. One of the limitations of this study was that information obtained from mothers having children aged less than 24 months is subject to recall bias. The study also shares the limitation of the cross-sectional study design.

## Conclusion

This study showed that four in ten infants were breastfed within the first hour of birth in Amibara district. Mothers who live in urban places, maternal attendances of formal education, giving birth by caesarean section and birth order of the index child were predictors of early initiation of breastfeeding. Thus efforts should be made to increase overall rates of female participation in formal education, provide breastfeeding education programs at the village level for girls and young women without formal education, and proper support and guidance of health care staffs during cesarean section delivery are important interventions to promote early initiation of breastfeeding in the study area.
